# Evolutionary Algorithm for RNA Secondary Structure Prediction Based on Simulated SHAPE Data

**DOI:** 10.1371/journal.pone.0166965

**Published:** 2016-11-28

**Authors:** Soheila Montaseri, Mohammad Ganjtabesh, Fatemeh Zare-Mirakabad

**Affiliations:** 1 Department of Computer Science, School of Mathematics, Statistics, and Computer Science, University of Tehran, Tehran, Iran; 2 Department of Computer Science, Faculty of Mathematics and Computer Science, Amirkabir University of Technology, Tehran, Iran; Bangladesh University of Engineering and Technology, BANGLADESH

## Abstract

**Background:**

Non-coding RNAs perform a wide range of functions inside the living cells that are related to their structures. Several algorithms have been proposed to predict RNA secondary structure based on minimum free energy. Low prediction accuracy of these algorithms indicates that free energy alone is not sufficient to predict the functional secondary structure. Recently, the obtained information from the SHAPE experiment greatly improves the accuracy of RNA secondary structure prediction by adding this information to the thermodynamic free energy as pseudo-free energy.

**Method:**

In this paper, a new method is proposed to predict RNA secondary structure based on both free energy and SHAPE pseudo-free energy. For each RNA sequence, a population of secondary structures is constructed and their SHAPE data are simulated. Then, an evolutionary algorithm is used to improve each structure based on both free and pseudo-free energies. Finally, a structure with minimum summation of free and pseudo-free energies is considered as the predicted RNA secondary structure.

**Results and Conclusions:**

Computationally simulating the SHAPE data for a given RNA sequence requires its secondary structure. Here, we overcome this limitation by employing a population of secondary structures. This helps us to simulate the SHAPE data for any RNA sequence and consequently improves the accuracy of RNA secondary structure prediction as it is confirmed by our experiments. The source code and web server of our proposed method are freely available at http://mostafa.ut.ac.ir/ESD-Fold/.

## Introduction

RNA molecules play vital roles in some cellular processes including genetic information carrier, biological catalysis and gene regulation [1]. The activity and function of non-coding RNAs are mainly related to their secondary structures formed by Watson-Crick and Wobble base pairs. In the two last decades, many algorithms and computational methods are proposed for the RNA secondary structure prediction based on maximizing the number of base pairs or minimizing the free energy.

RNAfold [2, 3] uses a dynamic programming algorithm to predict minimum free energy (MFE) structures as well as to compute partition functions and base pairing probabilities. UNAfold [4] combines free energy minimization, partition function calculation and stochastic sampling to predict RNA secondary structure using dynamic programming algorithm. MFold [5] also uses dynamic programming approach to predict RNA secondary structure based on energy minimization method [6]. RNAstructure [7] predicts and analyzes RNA secondary structure using thermodynamics and nearest neighbor parameters.

Although MFE is known as a suitable physical measure for RNA secondary structure prediction, the results show that this criterion does not have enough ability to predict the correct structure.

Recently, a new technology entitled Selective 2′-Hydroxyl Acylation analyzed by Primer Extension (SHAPE) significantly improves the consistency between the natural and predicted structures [8–10]. SHAPE technology could extract the reactivity information of almost all nucleotides in an RNA sequence, calculated by chemical experiment methods as well as ShapeFinder software [11]. For each RNA sequence, this experiment should be performed to find its SHAPE reactivities. These SHAPE reactivities are then converted to pseudo-free energy for increasing the prediction accuracy by adding them to the free energy function. Several attempts have been done to overcome the existing noises in the experimental SHAPE data. A bootstrapping method [9] was presented to estimate the variance and helix-by-helix confidence levels of predicted secondary structures based on resampling the measured SHAPE data. Jacknife approach [12] was also proposed to estimate the influence of a given experimental data set on SHAPE-directed RNA secondary structure modeling. In this method, by discarding 35% of the data, the confidence levels of SHAPE-directed RNA secondary structure prediction were significantly higher than those calculated by bootstrapping. After performing the SHAPE experiment and producing the data, the SHAPE reactivities are converted to pseudo-free energies and used in RNA structure prediction methods. Low et al. [13] composed SHAPE data with the nearest-neighbor thermodynamic rules for the RNA structure prediction based on dynamic programming algorithm implemented in RNAstructure method. GTFold [14] is a parallel version of RNAstructure. This method reports one structure that minimizes the summation of free energy and SHAPE pseudo-free energy as the best predicted RNA secondary structure. The main limitation of these methods is that the experimental SHAPE data should be available for the given RNA sequences. To overcome this limitation, Sükösd et al. [10] simulated SHAPE data for RNA sequences by estimating some probability distribution functions for the nucleotides appeared in unpaired, stacked and helix-end regions using the available SHAPE data on 16S rRNA and 23S rRNA. Even in this method, to simulate SHAPE data for an RNA sequence, its secondary structure is required to be able to decompose the given sequence into the mentioned regions.

In this paper, a new evolutionary algorithm is proposed to predict RNA secondary structure based on simulated SHAPE data. Since the RNA secondary structure is not available for the given RNA sequence, a population of RNA secondary structures is generated using a base pair probability matrix, which is constructed to represent the pairing probability of each pair of nucleotides. This population could help us to decompose the given RNA sequence into unpaired, stacked and helix-end regions with respect to the generated structures, and simulate the SHAPE data for the nucleotides appeared in these regions. Then, all the structures in the current population are improved using the evolutionary method. The fitness value of each structure is considered as the summation of free energy and SHAPE pseudo-free energy values. The new population is constructed using the best solutions among the previous population and improved individuals. This process is continued until the best fitness value remains constant during the two last populations. Finally, the structure corresponding to the best fitness value is reported as output. The proposed algorithm is performed on two real datasets of RNA sequences. The first dataset contains fifteen standard RNA sequences with their available SHAPE data including *Adenine-riboswitch*, *tRNA-Asp-Yeast*, *tRNA-Phe*, *MDLOOP*, *HCV IRES domain2*, *5S-Ecoli*, *ADDRSW*, *c-id-GMP-riboswitch*, *RNASEP-B.subtilis*, *p546*, *5srRNA*, *Glycine-riboswitch*, *TRP4*, *16S rRNA* and *23S rRNA* [15]. The second dataset including available families of RNA-STRAND dataset named NDB, PDB, RFA, SPR, SRP, ASE, TMR and CRW [16], in which SHAPE data is not available. The validity and accuracy of our algorithm are investigated and compared with the *RNAstructure* method which supports RNA SHAPE data.

In Section 2, after providing several preliminaries, the details of our proposed method are described. The results of our method as well as its comparison with the other methods are shown and discussed in Section 3. Finally, the conclusion is provided in Section 4.

## Materials and Methods

An RNA sequence is composed of four nucleotides, namely Adenine (*A*), Cytosine (*C*), Guanine (*G*), and Uracil (*U*) which can be considered as a string over *Σ*, where *Σ* = {*A*, *C*, *G*, *U*}. An RNA sequence is folded to itself and some base pairs are formed by the creation of hydrogen bonds between complementary bases. This set of base pairs is known as RNA secondary structure. The simplest form of RNA secondary structure is pseudoknot free which is formally defined as follows. An RNA (pseudoknot free) secondary structure *ω* for the RNA sequence *δ* = *δ*_1_ *δ*_2_⋯*δ*_*n*_ of length *n* is a collection of base pairs (*δ*_*i*_, *δ*_*j*_), where for any two base pairs (*δ*_*i*_, *δ*_*j*_) and (*δ*_*s*_, *δ*_*t*_) in *ω*, *i* = *s* ⇔ *j* = *t* and either *i* < *s* < *t* < *j* or *i* < *j* < *s* < *t* holds.

The RNA secondary structure contains some structural components including stems, hairpin loops, bulge loops, internal loops, multi loops, and external loops. A stem in the RNA secondary structure is a set of consecutive base pairs, while the different loops indicate unpaired regions or regions of non-canonical pairs.

The thermodynamic free energy of an RNA secondary structure is assumed to be the sum of its energies associated to its structural components. In other words, the free energy of an RNA sequence *δ* over the structure *ω*, denoted by *E*(*δ*, *ω*), is the sum of free energies associated to the stems (Δ*G*_*stems*_) and loops (Δ*G*_*loops*_) as written in Eq (1).

$$\begin{array}{c} E\left(\delta ,\omega \right)=\sum \Delta G_{stems}+\sum \Delta G_{loops}. \end{array}$$ (1)

The free energy of a stem is defined as the sum of free energies associated to its adjacent base pairs. The free energy of a loop is mainly dependent on its length, the start and end mismatch of the loop, and specified sub-sequence [14]. For a given RNA sequence, the secondary structure is usually predicted based on minimizing free energy obtained by the nearest neighbor thermodynamic model [17, 18]. In order to improve the prediction accuracy of RNA structure prediction, SHAPE data is added to the free energy as pseudo-free energy. The derived pseudo-free energy of SHAPE value for *i*th nucleotide is calculated according to the following equation:

$$\begin{array}{c} \Delta G_{i}=m\times \ln\left(SR(i)+1\right)+b, \end{array}$$ (2)

where *SR*(*i*) shows the SHAPE reactivity value for *i*th nucleotide in the RNA. The parameters *m* and *b* scale the strength of experimental contribution to the energy function. The intercept *b* represents pseudo-free energy contribution of a paired nucleotide, whose SHAPE reactivity is zero. The slope *m* shows the strength of the energetic penalty assigned for paired nucleotides with high SHAPE reactivities. Default values for parameters *m* and *b* are set to 2.6*kcal*/*mol* and −0.8*kcal*/*mol*, respectively [13].

Based on the evolutionary algorithm, we propose a new method for predicting the RNA secondary structure that uses both free energy and SHAPE pseudo-free energy values. The main steps of our proposed method, entitled *ESD-Fold*, are depicted in Algorithm 1. More details about these steps are provided in the following sub-sections.

**Algorithm 1** Evolutionary algorithm for RNA secondary structure prediction based on simulated SHAPE data.

**ESD-Fold** (RNA sequence *δ*)

 1〉   *S*_*δ*_← All potential stems in *δ*

 2〉   *P*[0]← The initial population of *m* secondary structures

 3〉   Evaluate (*P*[0])

 4〉   *best*[0]←*Argmin*_*ω*_{*fitness*(*ω*)|*ω* ∈ *P*[0]}

 5〉   *i* ← 0

 6〉   **do**

 7〉     **for each** *ω* ∈ *P*[*i*] **do**

 8〉       *ω*′← modify *ω* by replacing one of its stems

 9〉       Add *ω*′ to the temporary population *Q*

10〉     **end for**

11〉     Evaluate (*Q*)

12〉     *P*[*i* + 1]← Select the *m* best solutions among *P*[*i*] and *Q*

13〉     *best*[*i* + 1]←*Argmin*_*ω*_{*fitness*(*ω*)|*ω* ∈ *P*[*i* + 1]}

14〉     *i* ← *i* + 1

15〉   **while** (*fitness*(*best*[*i*]) ≠ *fitness*(*best*[*i* − 1]))

16〉   **return** (*best*[*i*])

  **Evaluate** (Population *P*)

17〉   **for each** *ω* ∈ *P* **do**

18〉     Simulate SHAPE data for *ω*

19〉     *fitness*(*ω*)←*E*(*δ*, *ω*) + *PE*(*δ*, *ω*)

20〉   **end for**

### Constructing all potential stems

In order to generate a population of secondary structures for the given RNA sequence, it is required to construct all potential stems (Line 1 of Algorithm 1). To do this, a base pair probability matrix is constructed for the given RNA sequence *δ* = *δ*_1_ *δ*_2_⋯*δ*_*n*_ as follows:

1. The *RNAfold* software is executed on *δ* to obtain the base pair probability matrix *P* = [*p*_*ij*_]_*n*×*n*_, where each element *p*_*ij*_ (0 ≤ *p*_*ij*_ ≤ 1) indicates the pairing probability between bases *δ*_*i*_ and *δ*_*j*_, where 1 ≤ *i* < *j* ≤ *n*. It should be noted that the RNAfold software is free of charge for academic users and available in address “http://rna.tbi.univie.ac.at/cgi-bin/RNAfold.cgi”.
2. Based on these probabilities, a potential stem *s* = <*r*_*s*_, *c*_*s*_, ℓ_*s*_, *p*_*s*_> is identified in which (*r*_*s*_, *c*_*s*_) shows the starting row and column of stem *s* in the matrix, ℓ_*s*_ represents the length of *s*, and *p*_*s*_ indicates the summation of base pair probabilities in *s* (*p*_*s*_ = ∑_(*i*, *j*)∈*s*_ *p*_*ij*_). The following conditions should be satisfied when identifying potential stems:
  $$\begin{array}{ccc} P[r_{s}+k,c_{s}-k]\neq 0 & & \forall k=0\cdots \ell_{s}-1, \end{array}$$ (3)
  $$\begin{array}{ccc} P[r_{s}-1,c_{s}+1]=0 & & if\;1<c_{s}\;\&\;r_{s}<n, \end{array}$$ (4)
  $$\begin{array}{ccc} P[r_{s}+\ell_{s},c_{s}-\ell_{s}]=0 & & if\;1\leq r_{s}-\ell_{s}\;\&\;c_{s}+\ell_{s}\leq n, \end{array}$$ (5)
3. Among the identified potential stems, those having length greater than or equal to three (ℓ_*s*_ ≥ 3) are selected and stored in the set *S*_*δ*_. By considering the length 3 as a cutoff, we eliminate all the stems of length 1 or 2 (less stable stems) from further processes. If not, these stems are more likely to be selected in the constructed structure that make it less stable. Therefore, this elimination helps us to produce more stable structures as well as to speed-up the running time of our algorithm.

**Fig 1:**
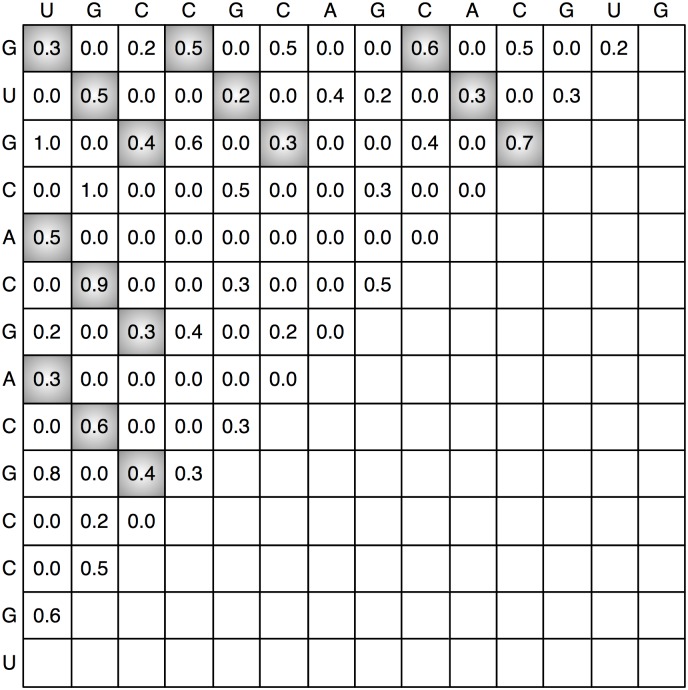
Base pair probability matrix.

As an example, Fig 1 represents the base pair probability matrix calculated for sequence *δ* = *GUGCACGACGCCGU*. In this figure, five different potential stems are indicated in gray and all the probabilities are presented with one decimal digit.

### Generating a population of RNA secondary structures

For generating the initial population *P*[0] of RNA secondary structures (Line 2 of Algorithm 1), a Roulette-Wheel method is employed. This Roulette-Wheel is constructed based on the assigned probability *p*_*s*_ to each potential stem *s*. Then, each secondary structure is formed by iteratively selecting stems from the Roulette-Wheel until *k* different stems (without any overlap) are obtained. During this process, if stem *s* overlaps with some stems in the structure, the overlapped segments are eliminated from *s* and the remaining segments are added to the structure. It is worth noting that these remaining segments might have small length (less than 3). According to this approach, the initial population *P*[0] of *m* (*m* is equal to the length of RNA sequence) secondary structures is generated. The appropriate value for the number of initial stems in each secondary structure (namely *k*) is considered as *n*/5. This value is obtained by analyzing the number of stems in the available structures taken from RNA-STRAND dataset [16] (see S1 File).

### Simulating SHAPE data

For simulating the SHAPE data corresponding to each RNA secondary structure *ω* in the current population (Line 18 of Algorithm 1), three different probability distribution functions are employed [10]. These probability distribution functions are computed by fitting a curve on available SHAPE data corresponding to nucleotides appeared in unpaired, stacked, and helix-end regions in 16*SrRNA* and 23*SrRNA* sequences [15].

### Computing fitness value

In order to evaluate the accuracy of the generated secondary structures, a fitness function is employed. This fitness function incorporates both free energy and pseudo-free energy values (Line 19 of Algorithm 1). Formally, the fitness value for the secondary structure *ω* is determined as follows:

$$\begin{array}{c} Fitness(\omega )=E(\delta ,\omega )+PE(\delta ,\omega ), \end{array}$$ (6)

where *PE*(*δ*, *ω*) is the SHAPE pseudo-free energy of the structure *ω* over the sequence *δ*. Analysis of the relationship between SHAPE data and thermodynamic free energy was previously done in [19]. This SHAPE pseudo-free energy is computed as:

$$\begin{array}{c} PE\left(\delta ,\omega \right)=2\times \sum_{\delta_{i}\in stacked}\Delta G_{i}+\sum_{\delta_{i}\in helix-end}\Delta G_{i}, \end{array}$$ (7)

where *stacked* and *helix–end* indicate the nucleotides appeared in stacked and helix-end regions of the structure, respectively.

### Evolutionary algorithm

For each secondary structure *ω* in the current population, an evolutionary algorithm is employed to improve the quality of that structure (Lines 6–15 of Algorithm 1). To this end, a stem is randomly selected from *ω* and it is replaced by another stem from *S*_*δ*_ to form a new structure *ω*′. The new stem is also selected from the potential stems using the constructed Roulette-Wheel. This process is iteratively continued until the following condition is satisfied:

$$\begin{array}{c} fitness\left(\omega^{\prime }\right)\geq \;fitness\left(\omega \right). \end{array}$$ (8)

Among the structures *ω* in the current population and the corresponding modified structures *ω*′, the number of *m* best structures (having lower fitness values) are selected to form the next population (Line 13 of Algorithm 1). The generation of new populations is continued until the best fitness value of the structures remains fixed during the two last populations (Lines 6-15 of Algorithm 1).

## Results

The evaluation of our proposed method is provided in this section and the results are compared with the other competitors. All methods have been executed on a machine with dual-Core Intel(R) Duo processor *T*6670 2.20 GHz and 4 GB of installed memory. A dataset containing fifteen RNA sequences with their available experimental SHAPE data is employed [15] in our first evaluation. This dataset includes *Adenine-riboswitch*, *tRNA-Asp-Yeast*, *tRNA-Phe*, *MDLOOP*, *HCV IRES domain2*, *5S-Ecoli*, *ADDRSW*, *c-id-GMP-riboswitch*, *RNASEP-B.subtilis*, *p546*, *5srRNA*, *Glycine-riboswitch*, *TRP4*, *16S rRNA* and *23S rRNA*. The prediction accuracy is calculated using Eq (9):

$$\begin{array}{c} Accuracy=(Sn+PPV)/2, \end{array}$$ (9)

where Sensitivity (*Sn*) and Positive Predictive Value (*PPV*) are defined as follows:

$$\begin{array}{ccc} Sn & = & TP/(TP+FN), \end{array}$$ (10)

$$\begin{array}{ccc} PPV & = & TP/(TP+FP). \end{array}$$ (11)

In the above formulas, *TP*, *FP* and *FN* indicate the number of correctly predicted base pairs, false predicted base pairs, and the unpredicted base pairs, respectively. Using this measure, we have compared the accuracies of our proposed method, RNAfold, and RNAstructure over this dataset. As presented in Table 1, the accuracies are provided for MFE RNAfold (Column 3), RNAstructure when it uses MFE (Column 4) and experimental SHAPE data (Column 5), as well as ESD-Fold when experimental and simulated SHAPE data are employed (Columns 6-7). The computational time (minutes) of ESD-Fold on this dataset is also shown in the last column of Table 1. Based on the average accuracies in this table, our method performs better than the other two competitors. For this dataset, two other measures are also employed to evaluate the performance of the ESD-Fold algorithm as presented in S1 File.

**Table 1:** Comparison of the results obtained by the MFE RNAfold (Column 3), and RNAstructure when it uses MFE (RNAstr. MFE, Column 4) and real SHAPE data (RNAstr. real, Column 5), and our proposed method (ESD-Fold) when the real and simulated SHAPE data are employed (real and simulated, Columns 6-7). The computational time (minutes) of ESD-Fold is represented in Column 8. Here, the values are rounded to one decimal digit.

RNA sequence	Length	RNAfold (MFE)	RNAstr. (MFE)	RNAstr. (real)	ESD-Fold (real)	ESD-Fold (simulated)	Comp. time (minutes)
Adenine	71	100	100	100	100	100	0.8
tRNA-Asp	75	88.4	73.9	73.9	93.1	88.6	0.9
tRNA-Phe	76	22	97.6	100	100	100	0.9
MDLOOP	80	91.7	87	100	100	100	0.9
HCV- domain2	95	57.4	88.3	88.3	73.2	77.5	1
5S-Ecoli	120	26.4	26	71.9	75.7	75.5	1.7
ADDRSW	121	100	95.7	95.7	100	100	1.3
CIDGMP	135	83.8	88	77	68	69.3	1.5
RNASEP4	154	69.9	50	75.7	71.8	71.8	1.9
p546	155	43.1	43.1	94.3	96.5	86	2.1
5srRNA	170	24.8	24.3	24.2	79.8	69.7	2.2
Glycine-riboswitch	198	62.7	88.1	88.1	95.5	77.9	2.4
TRP4P6	202	77.7	87.3	86.14	84.9	84.9	2.9
16S rRNA	1542	40.6	35.1	74.3	46.5	48.7	54.7
23S rRNA	2904	47	46.3	63.6	49.1	40.8	204
**Average**		**62.4**	**68.7**	**80.9**	**82.3**	**79.4**	

As it is observed in Table 1, the accuracy of ESD-Fold is decreased for some RNA sequences such as *HCV- domain2* and *CIDGMP*. The accuracy of RNAstructure is also decreased for these sequences when the SHAPE data is utilized. Therefore, it seems that the experimental or simulated SHAPE data for these sequences could not improve the accuracy.

The accuracies of ESD-Fold are also decreased for longer sequences, namely *16S rRNA* and *23S rRNA*. Although, longer potential stems are selected with higher probability (using Roulette-Wheel selection mechanism), the number of stems is also important. For longer RNA sequence, the number of shorter potential stems is huge and these stems are more likely to be selected. Therefore, the overlapped segment of longer potential stems are eliminated and the remaining shorter segments (that are less stable) are considered as potential stems. This obviously decreases the accuracy of ESD-Fold for longer RNA sequences.

In order to calculate the other statistics, namely TP, FP, FN, Sn, PPV, Accuracy, and the number of generations, we have performed 100 independent executions of our proposed method over the fifteen RNA sequences. The obtained values for these statistics are provided in Table 2.

**Table 2:** The average values of TP, FP, FN, Sn, PPV, Accuracy ± standard deviation (Acc ± sd), and number of generations for 100 independent executions of the proposed method over the fifteen RNA sequences. Here, the values are rounded to two decimal digits.

RNA sequence	TP	FP	FN	Sn	PPV	Acc ± sd	Num. of Generations
Adenine	21	0	0	1	1	1	3.18
tRNA-Asp	17.98	4.02	0.23	0.82	0.99	0.9 ± 0.03	3.23
tRNA-Phe	20.77	0.23	0.12	0.99	0.99	0.99 ± 0.02	3.33
MDLOOP	10	0	0	1	1	1	3.06
HCV- domain2	17.81	8.19	3.06	0.69	0.86	0.77 ± 0.05	3.28
5S-Ecoli	19.86	15.14	7.04	0.57	0.74	0.65 ± 0.17	3.17
ADDRSW	20.96	0.04	0.11	1	1	1 ± 0.01	2.96
CIDGMP	20.32	4.68	8.09	0.81	0.72	0.77 ± 0.03	3.34
RNASEP4	32.55	12.45	5.21	0.72	0.86	0.79 ± 0.08	3.21
p546	48.51	9.49	5.81	0.84	0.89	0.86 ± 0.1	3.4
5srRNA	16.65	17.35	7.77	0.49	0.72	0.6 ± 0.1	2.84
Glycine-riboswitch	32.96	7.04	9.19	0.82	0.78	0.8 ± 0.12	3.3
TRP4P6	43.76	4.24	9.27	0.92	0.83	0.87 ± 0.01	3.03
16S rRNA	212.26	244.74	158.47	0.46	0.57	0.52 ± 0.01	3.47
23S rRNA	346.59	483.41	483.41	0.42	0.55	0.48 ± 0.01	3.44
**Average**				**0.77**	**0.83**	**0.8 ± 0.05**	

**Fig 2:**
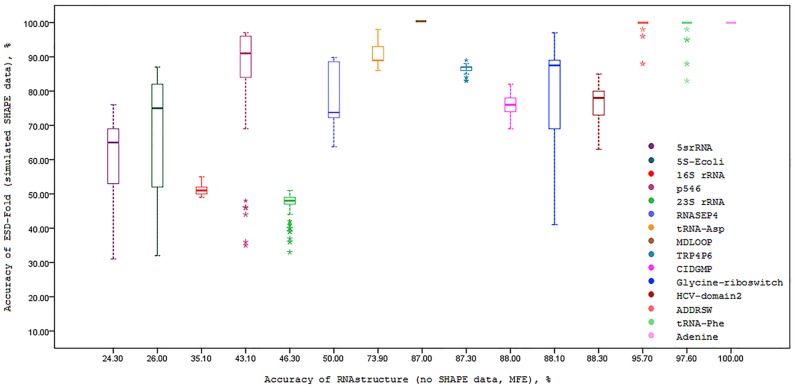
Boxplots of simulated SHAPE data prediction accuracy versus accuracy of the MFE prediction of RNAstructure software. In each box, the midline marks the median accuracy for 100 predictions.

**Fig 3:**
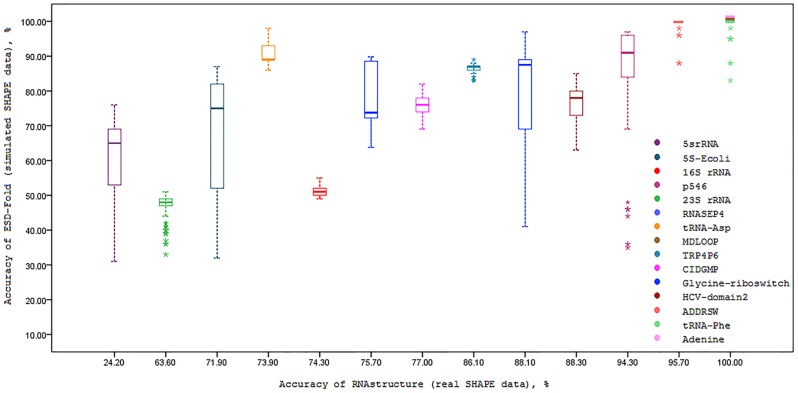
Boxplots of simulated SHAPE data prediction accuracy versus accuracy of the real SHAPE prediction of RNAstructure software. In each box, the midline marks the median accuracy for 100 predictions.

**Fig 4:**
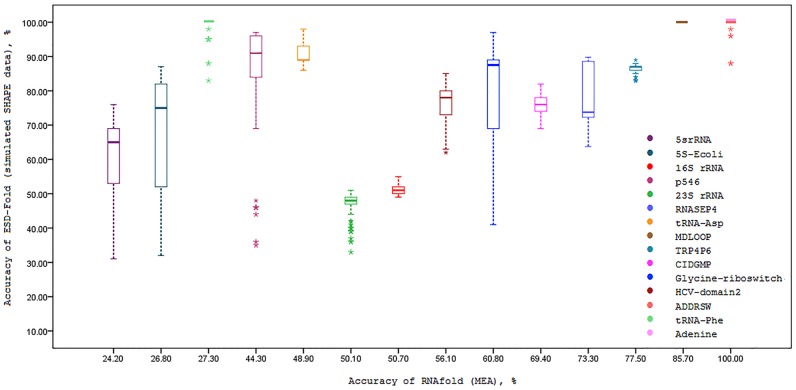
Boxplots of simulated SHAPE data prediction accuracy versus accuracy of MEA structure of RNAfold software. In each box, the midline marks the median accuracy for 100 predictions.

We also investigated the effect of simulated SHAPE data on prediction accuracy for each fifteen RNA sequences. To this end, the correlation between the prediction accuracies of ESD-Fold (for 100 independent executions for each sequence) and MFE RNAstructure (no SHAPE data) are calculated and presented in Fig 2. As it is understood from this figure, using ESD-Fold with simulated SHAPE data greatly improves the prediction accuracy of RNA secondary structure. The same analysis is performed for the case of RNAstructure (real SHAPE data) as well as maximum expected accuracy (MEA) of RNAfold method. The results of these analysis are presented in Figs 3 and 4, respectively. The effect of simulated SHAPE data on individual base pairs is also analysed. Let *M* and *D* respectively denote the set of base pairs predicted by MFE RNAstructure (no SHAPE data) and ESD-Fold (using simulated SHAPE data). The PPV of the MFE structure *M* is calculated for each RNA sequence as shown in column 2 of Table 3. For each RNA sequence, the PPVs corresponding to the subsets *M* ∩ *D* (common base pairs) and *M*∖*D* (remaining MFE base pairs) for 100 independent executions of ESD-Fold are also calculated (Table 3, columns 3 and 4, respectively). As it is observed in this table, the average PPV of the common base pairs (*M* ∩ *D*) is high for each sequence. Likewise, the average PPV of the remaining MFE base pairs (*M*∖*D*) is low. The higher PPV for common base pairs indicates the great impact of simulated SHAPE data in the RNA secondary structure prediction.

**Table 3:** PPV(M) shows the number of native base pairs in the MFE structure M. PPVs corresponding to the sets of common base pairs and remaining MFE base pairs were computed. Values in columns 3 and 4 are the average of 100 independent executions of ESD-Fold ± standard deviation. Here, the values are rounded to two decimal digits.

RNA sequence	PPV(M)	PPV(M ∩ D)	PPV(M∖D)
Adenine	1	1	0
tRNA-Asp	0.76	0.99 ± 0.01	0.32 ± 0.18
tRNA-Phe	1	1	0.13 ± 0.23
MDLOOP	0.71	1	0
HCV- domain2	0.32	1	0
5S-Ecoli	0.25	0.89 ± 0.15	0 ± 0.01
ADDRSW	0.91	1	0.01 ± 0.02
CIDGMP	0.8	0.81 ± 0.04	0.77 ± 0.1
RNASEP4	0.5	0.96 ± 0.06	0.08 ± 0.06
p546	0.44	0.97 ± 0.05	0.04 ± 0.06
5srRNA	0.23	0.68 ± 0.15	0
Glycine-riboswitch	0.84	0.9 ± 0.05	0.31 ± 0.3
TRP4P6	0.83	0.92 ± 0.02	0.44 ± 0.06
16S rRNA	0.34	0.77 ± 0.07	0.08 ± 0.02
23S rRNA	0.44	0.77 ± 0.02	0.23

Our proposed method is also applied on RNA sequences taken from the RNA-STRAND dataset, where no SHAPE data is available for them. This dataset includes eight families of RNA-STRAND, namely *NDB*, *PDB*, *RFA*, *SPR*, *SRP*, *ASE*, *TMR* and *CRW*, where for each family, the first ten sequences (according to their identification numbers) are selected. Column 2 in Table 4 shows the average length of the selected sequences for each family. Our method, RNAfold and RNAstructure are applied on this dataset and the average accuracies for each family are depicted in Table 4 (Columns 3, 4 and 5, respectively). The average computational time of the proposed method on this dataset is also presented in the last column of this table. As it can be seen, our method predicts RNA secondary structures with higher accuracy compared to the other competitors.

**Table 4:** Comparison of the results obtained by the MFE RNAfold (Column 3), and RNAstructure when it uses MFE (Column 4), and our proposed method (ESD-Fold) when the simulated SHAPE data is employed (Column 5). The average of computational time (minutes) of ESD-Fold on each family is shown in Column 6. Here, the values are rounded to one decimal digit.

RNA sequence	Length	RNAfold (MFE)	RNAstr. (MFE)	ESD-Fold (simulated)	Comp. time (minutes)
NDB	16	100	100	100	0.3
PDB	32	80.7	46.1	83.6	0.5
RFA	54	86.3	86.2	92.4	1
SPR	76	72.6	89.4	96.4	1
SRP	206	65.4	68.9	68.5	3.8
ASE	302	50.1	51.8	54.8	9.2
TMR	358	33.1	46	41.6	7.2
CRW	752	41.3	53.4	56.8	34
**Average**		**66.2**	**67.7**	**74.2**	

All the previously mentioned statistics are also computed for this dataset, using 200 executions of ESD-Fold for each family (20 executions per each RNA sequence), and presented in Table 5.

**Table 5:** The average values of TP, FP, FN, Sn, PPV, Accuracy ± standard deviation (Acc ± sd), and number of generations for 200 independent executions of the proposed method over each 8 families of the RNA-STRAND dataset. Here, the values are rounded to two decimal digits.

RNA sequence	TP	FP	FN	Sn	PPV	Acc ± sd	Num. of Generations
NDB	6	0	0	1	1	1	3.62
PDB	8.21	2.7	0.16	0.77	0.97	0.87 ± 0.12	2.98
RFA	14.2	0.8	2.3	0.95	0.86	0.9 ± 0.03	3.06
SPR	20.49	0.42	1.22	0.98	0.95	0.96 ± 0.04	3
SRP	36.11	8.6	9.71	0.72	0.7	0.71 ± 0.07	2.88
ASE	50.16	40.75	41.73	0.56	0.55	0.56 ± 0.07	2.97
TMR	41.19	49.92	52.27	0.5	0.44	0.47 ± 0.07	3.09
CRW	168.95	148.51	158.17	0.59	0.56	0.58 ± 0.08	3.09
**Average**				**0.76**	**0.76**	**0.76 ± 0.08**	

## Conclusions

As it is mentioned in the manuscript, the SHAPE data could not be simulated for an RNA sequence without knowing its secondary structure. The main achievement of our proposed method is to computationally simulate the SHAPE data for a given sequence with secondary structure obtained by using an evolutionary algorithm. To do this, a population of RNA secondary structures is generated and employed in the SHAPE data simulation phase. These structures are further improved by an evolutionary method. The results show the high accuracy and efficiency of the proposed method in comparison to the RNAstructure.

In our proposed method, a matrix representation of pairing probabilities of each two nucleotides in an RNA sequence was created. First of all, a population of RNA secondary structures for the given RNA sequence is constructed based on Roulette-Wheel selection. Then, SHAPE data was simulated for each secondary structure in the population. Here, the fitness value for each structure was considered as summation of free energy and pseudo-free energy values. Afterwards, an evolutionary algorithm was applied on each secondary structure in the population to improve it. Generation of population is continued until the best structure in the two last populations remains constant. Finally, the structure with minimum summation of free and pseudo-free energies is reported as the predicted RNA structure.

As it is mentioned in the results, the accuracy of ESD-Fold is not high enough for longer RNA sequences. One direction to further improve the ESD-Fold is to find a mechanism for selecting the stable stems with higher probability prior to those stems that are less stable, independent of the number of these stems. This would enable the ESD-Fold to produce more accurate structures, specially for longer sequences.

## Supporting Information

S1 File ESD-Fold: RNA Folding Based on Simulated SHAPE Data. (PDF)
