# Supplementary material for: Evolutionary Algorithm for RNA Secondary Structure Prediction Based on Simulated SHAPE Data
Source: PLoS One. 2016 Nov 28;11(11):e0166965. doi: 10.1371/journal.pone.0166965 (PMC5125645; doi:10.1371/journal.pone.0166965)
Supplement: S1 File — (PDF) [file pone.0166965.s001.pdf]

Supporting Information

ESD-Fold: RNA Folding Based on Simulated SHAPE Data

Authors: Soheila Montaseri[1], Mohammad Ganjtabesh[1*], Fatemeh Zare-Mirakabad[2]

Affiliations:
1 Department of Computer Science, School of Mathematics, Statistics, and Computer Science, University of Tehran, Tehran, Iran.
2 Department of Computer Science, Faculty of Mathematics and Computer Science, Amirkabir University of Technology, Tehran, Iran.

*Correspondence to: mgtabesh@ut.ac.ir

1 Further evaluation of ESD-Fold

Besides the accuracy value, we have considered two other metrics, namely Matthews Correlation Coefficient (MCC) [1] and F-measure (F) [2], to evaluate the performance of ESD-Fold algorithm. These metrics are computed using sensitivity (Sn) and positive predictive value (PPV) as follows:

$$MCC = \frac{TP \times TN - FP \times FN}{\sqrt{(TP + FP)(TP + FN)(TN + FP)(TN + FN)}} \tag{1}$$

$$F = \frac{2 \times Sn \times PPV}{Sn + PPV} \tag{2}$$

It was also indicated in [1] that $\sqrt{Sn \times PPV}$ is a good approximation for MCC. Using elementary calculus, one can show that the values of MCC and F are less than or equal to the accuracy value. Since no significant differences are observed for this measures (see Table S1), we only used accuracy measure in our manuscript. The average values of accuracy, MCC and F for ESD-Fold (simulated SHAPE data) on fifteen RNA sequences over 100 trials are provided in Table S1.

Table S1. The average values of accuracy, MCC and F for ESD-Fold on the first dataset over 100 trials. Here, the values are rounded to two decimal digits.

RNA sequence	Accuracy	MCC	F
Adenine	1	1	1
tRNA-Asp	0.9	0.9	0.9
tRNA-Phe	0.99	0.99	0.99
MDLOOP	1	1	1
HCV-domain2	0.77	0.77	0.76
5S-Ecoli	0.65	0.65	0.64
ADDRSW	1	1	1
CIDGMP	0.77	0.77	0.77
RNASEP4	0.79	0.79	0.79
p546	0.86	0.86	0.86
5srRNA	0.6	0.59	0.58
Glycine-riboswitch	0.8	0.8	0.8
TRP4P6	0.87	0.87	0.87
16S	0.52	0.52	0.51
23S	0.48	0.48	0.48
Average	0.8	0.8	0.8

2 Selecting the number of initial stems in RNA secondary structure

The number of initial stems, k, in each secondary structure is chosen to be $n/5$, where n is the length of the given RNA sequence. To find out the number of appropriate stems in each structure, we performed as follows. First, we counted the number of stems (with different size) for all RNA sequences taken from RNA-STRAND dataset. Then, we eliminated those stems that are short (have length less than or equal to 2). After that, the weighted average of stem lengths is computed as 5.35. For this reason, we considered $n/5$ as the number of initial stems in the population. The length of each stem and the number of stems of that length are presented in Table S2.

Table S2. The length of stems and the number of stems of that length for RNA sequences from RNA-STRAND dataset.

Length of stem	The number of stems
1	10067
2	21149
3	22643
4	18129
5	11505
6	10166
7	8534
8	6709
9	1935
10	2922
11	729
12	1033
13	275
14	124
15	80
16	83
17	51
18	78
19	21
20	32
21	41
22	37
23	7
24	14
25	54
26	14
27	46
28	20
29	2
30	3
32	1
33	1
34	1
36	1
37	1
41	1
43	1
265	1
276	1
412	1

References

1. Seemann SE, Richter AS, Gesell T, Backofen R, Gorodkin J. PETcofold: predicting conserved interactions and structures of two multiple alignments of RNA sequences, Bioinformatics. 2011;2:211-219.

2. Kato Y, Akutsu T, Seki H. A grammatical approach to RNA-RNA interaction prediction, Pattern Recognition. 2009;42:531-538.
